# Brain myoinositol as a potential marker of amyloid-related pathology: A longitudinal study

**DOI:** 10.1212/WNL.0000000000006852

**Published:** 2019-01-29

**Authors:** Olga Voevodskaya, Konstantinos Poulakis, Pia Sundgren, Danielle van Westen, Sebastian Palmqvist, Lars-Olof Wahlund, Erik Stomrud, Oskar Hansson, Eric Westman, for the Swedish BioFINDER Study Group

**Affiliations:** From the Division of Clinical Geriatrics (O.V., K.P., L.-O.W., E.W.), Department of Neurobiology, Care Sciences and Society, Karolinska Institute, Stockholm; Department of Diagnostic Radiology (P.S., D.v.W.), Lund University; Imaging and Function (D.v.W.), Skåne University Health Care, Lund; Clinical Memory Research Unit (S.P., E.S., O.H.), Department of Clinical Sciences, Malmö, Lund University; Memory Clinic (E.S., O.H.), Skåne University Hospital, Malmö, Sweden; and Department of Neuroimaging (E.W.), Centre for Neuroimaging Sciences, Institute of Psychiatry, Psychology and Neuroscience, King's College London, UK.

## Abstract

**Objective:**

To investigate the association between longitudinal changes in proton magnetic resonance spectroscopy (MRS) metabolites and amyloid pathology in individuals without dementia, and to explore the relationship between MRS and cognitive decline.

**Methods:**

In this longitudinal multiple time point study (a subset of the Swedish BioFINDER), we included cognitively healthy participants, individuals with subjective cognitive decline, and individuals with mild cognitive impairment. MRS was acquired serially in 294 participants (670 individual spectra) from the posterior cingulate/precuneus. Using mixed-effects models, we assessed the association between MRS and baseline β-amyloid (Aβ), and between MRS and the longitudinal Mini-Mental State Examination, accounting for *APOE*, age, and sex.

**Results:**

While baseline MRS metabolites were similar in Aβ positive (Aβ+) and negative (Aβ−) individuals, in the Aβ+ group, the estimated rate of change was +1.9%/y for myo-inositol (mI)/creatine (Cr) and −2.0%/y for *N*-acetylaspartate (NAA)/mI. In the Aβ− group, mI/Cr and NAA/mI yearly change was −0.05% and +1.2%; however, this was not significant across time points. The mild cognitive impairment Aβ+ group showed the steepest MRS changes, with an estimated rate of +2.93%/y (*p* = 0.07) for mI/Cr and −3.55%/y (*p* < 0.01) for NAA/mI. Furthermore, in the entire cohort, we found that Aβ+ individuals with low baseline NAA/mI had a significantly higher rate of cognitive decline than Aβ+ individuals with high baseline NAA/mI.

**Conclusion:**

We demonstrate that the longitudinal change in mI/Cr and NAA/mI is associated with underlying amyloid pathology. MRS may be a useful noninvasive marker of Aβ-related processes over time. In addition, we show that in Aβ+ individuals, baseline NAA/mI may predict the rate of future cognitive decline.

Accumulation of β-amyloid (Aβ) in the brain is the main hallmark of Alzheimer disease (AD), and can be detected by analyzing CSF Aβ_42_ levels. In AD, CSF Aβ_42_ might change before Aβ fibrils become detectable with PET,^1^ and is the earliest disease biomarker to become abnormal.^2^ Magnetic resonance spectroscopy (MRS) is a noninvasive technique that allows quantifications of specific brain metabolites in vivo.

Recent MRS studies provide evidence of myo-inositol (mI) being the most relevant MRS metabolite in AD.^3–5^ Specifically, elevated mI seems to be related to Aβ plaque pathology—higher baseline levels of mI are associated with increased Aβ accumulation over time.^5^ Another MRS metabolite consistently changed in dementia is the neuronal marker *N*-acetylaspartate (NAA).

In this study, we investigate the association between longitudinal changes in the ratios mI/creatine (Cr) and NAA/mI and amyloid pathology. MRS measurements from a single voxel located in the posterior cingulate cortex (PCC)/precuneus were acquired serially from 294 participants at baseline and 2- and 4-year follow-up. Using information on the presence of amyloid pathology and the genetic risk factor *APOE* ε4, we aimed to investigate the extent to which the rates of change in MRS metabolites mI/Cr and NAA/mI are associated with Aβ pathology. Furthermore, we explored whether baseline levels of NAA/mI were able to predict future cognitive decline.

## Methods

### Standard protocol approvals, registrations, and patient consents

All participants gave written consent to participate in the study. Ethical approval was given by the ethical committee of Lund University, Sweden.

### Participants

This study only contained data from individuals without dementia (characterized as cognitively normal [CTL]), individuals with subjective cognitive decline (SCD), or those with mild cognitive impairment (MCI). The participants stemmed from the Swedish BioFINDER (Biomarkers for Identifying Neurodegenerative Disorders Early and Reliably) Study (detailed information available at biofinder.se).

The CTL subset included participants based on the following characteristics: (1) baseline age 60 years or older, (2) 28–30 Mini-Mental State Examination (MMSE) points at the time of the screening, (3) no subjective cognitive impairment, and (4) fluent Swedish. Participants were excluded if there was evidence of significant neurologic or psychiatric disease, MCI, or dementia.

Participants with SCD and MCI were enrolled from 3 outpatient memory clinics. Individuals were eligible if they: (1) were referred to the memory clinic because of cognitive impairment, (2) did not fulfill the criteria for any dementia disorder, (3) had an MMSE of 24–30 points at the time of screening, (4) were 60 to 80 years old, and (5) were fluent in Swedish. Participants were excluded if (1) the cause of their cognitive impairment was clearly other than prodromal dementia, (2) they had significant somatic disease, or (3) they refused to undergo neuropsychological assessment or lumbar puncture. The classification into SCD and MCI was based on a comprehensive battery of neuropsychological tests as well as an evaluation by a senior neuropsychologist.

In the current study, only participants with baseline CSF and high-quality MRS data were eligible. A total of 294 participants were investigated: healthy controls (n = 137) from cohort 1, and patients with SCD (n = 83) and patients with MCI (n = 74) from cohort 2.

### MRS acquisition and analysis

MRS was performed on a 3-tesla Siemens TrioTim scanner (Siemens Medical Solutions, Malvern, PA), using the point-resolved spectroscopy single-voxel sequence, at an echo time of 30 milliseconds (ms) and a repetition time of 2,000 ms. The 2 × 2 × 2 cm^3^ voxel was situated in the midsagittal PCC/precuneus region. This area has been recommended as the appropriate region for MRS in AD,^6^ and has frequently been the region of choice by previous large-scale MRS studies.^5,7,8^ Metabolite levels were quantified relative total creatine (creatine + phosphocreatine, Cr) concentration, due to the relative stability of the resonance peak of Cr.^9^ Another marker routinely examined using MRS is choline (Cho). Although the significance of Cho changes for AD pathology is unclear, we present these data briefly in the report.

MRS spectra were processed using the LCmodel^10,11^ software and inspected for artifacts and quality. A total of 214 individual spectra were excluded from the analysis. Individual spectra were excluded because of insufficient quality; individuals were excluded when no baseline spectrum was available, as well as when spectra from only one time point existed (flowchart data available from Dryad, Additional Methods, doi.org/10.5061/dryad.rd681sp). The mean FWHM (full width at half maximum) of the remaining spectra was 6.6 Hz (SD = 1.6 Hz); mean signal-to-noise ratio was 24 (SD = 4.5). LCModel uses estimated SDs (Cramér-Rao lower bounds) as reliability indicators for metabolite concentrations; the value of %SD <20% is often used as a limit for acceptable reliability. However, metabolites mI, NAA, and Cho are readily detectable at echo time = 30 ms; for mI/Cr, NAA/Cr, and Cho/Cr, %SD was ≤7% for all spectra.

### CSF acquisition and analysis

The procedure of CSF collection and analysis followed the Alzheimer's Association flowchart for CSF biomarkers.^12,13^ Lumbar CSF samples were collected at 3 centers, stored in polypropylene tubes at −80°C and analyzed at the same time using 2 ELISAs. CSF Aβ_42_ was analyzed by INNOTEST ELISAs (Fujirebio Europe, Ghent, Belgium).^14^ CSF Aβ_42_ concentration ≤530 ng/L was considered abnormal, as previously published.^4^

### Statistical procedures

Cross-sectional between-group differences in MRS data were assessed using analysis of variance, accounting for age, sex, and *APOE* ε4 carriership, and Tukey honestly significant difference test for post hoc analyses.

Longitudinal mI/Cr and NAA/mI changes were analyzed using linear mixed-effects models with subject-specific intercepts. Fixed effects were selected depending on the primary research question. In model 1, the fixed effects were baseline CSF Aβ group (normal/abnormal), visit number, and the Aβ group-by-visit interaction. In model 2, the fixed effects were baseline clinical diagnosis, visit number, and the diagnosis-by-visit interaction. Models 1 and 2 were created using data from all participants. Model 3 was analogous to model 1, while only using data from patients with MCI. Models 1–3 took into account baseline age, sex, and *APOE* ε4 carriership. Annualized percentual changes in MRS measures were derived from the model estimates by taking the average of the estimated rates of metabolite concentration change between visits 1 and 2, and visits 1 and 3. The interaction term *APOE* × visit was not included in the final models, since it was found to be insignificant in all models for both outcome variables mI/Cr and NAA/mI.

A similar approach was used in the analysis of longitudinal cognitive data. Here, we modeled the change in MMSE score over time as a function of baseline NAA/mI values. The fixed effects were baseline NAA/mI (high/low), visit number, and the NAA/mI-by-visit interaction. When modeling cognitive decline over time, we took into account age, sex, years of education, and *APOE* ε4 status.

Since the *APOE* ε2 allele may have a neuroprotective effect of the ε2 allele, 8 participants with genotype ε2/ε4 were excluded from the analyses.

Statistical analyses were performed using R (R Foundation for Statistical Computing, Vienna, Austria, r-project.org).

### Data availability

Anonymized data will be shared by request from any qualified investigator for the sole purpose of replicating procedures and results presented in the report provided that data transfer is in agreement with EU legislation on the general data protection regulation.

## Results

### Study sample

**Figure 1:**
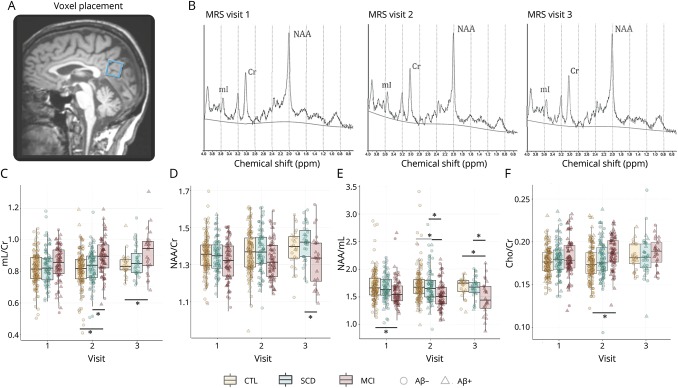
Voxel placement, serial MRS acquisition, and metabolite levels across diagnoses and biomarker groups (A) Example of an MRS voxel placement and (B) acquired serial spectra at baseline, visit 1, and visit 2, from a 73-year-old cognitively healthy woman. (C–F) Serial spectroscopic data across diagnostic groups. (C) mI/Cr ratio levels, (D) NAA/Cr ratio levels, (E) NAA/mI ratio levels, (F) Cho/Cr ratio levels. Significant between-group differences (reported at *p < 0.05) were obtained using analysis of variance, accounting for age, sex and *APOE* ε4 carriership, with Tukey honestly significant difference tests for post hoc comparisons. Aβ+ = CSF Aβ_42_ ≤530 ng/L; Aβ− = CSF Aβ_42_ >530 ng/L. Aβ = β-amyloid; Cho = choline; Cr = creatine; CTL = cognitively normal control; MCI = mild cognitive impairment; mI = myo-inositol; MRS = magnetic resonance spectroscopy; NAA = *N*-acetylaspartate; SCD = subjective cognitive decline.

A total of 670 individual spectra were acquired, averaging approximately 2.2 spectra per individual for CTL, 2.4 for SCD, and 2.3 for MCI. The timing and number of spectra are presented in table 1. Demographic details of the diagnostic groups are presented in table 2. As expected, there were differences in cognitive function across the 3 diagnostic groups both at baseline and follow-up (table 2). Voxel placement and an example of serially acquired spectra are presented in figure 1, A and B.

**Table 1:**
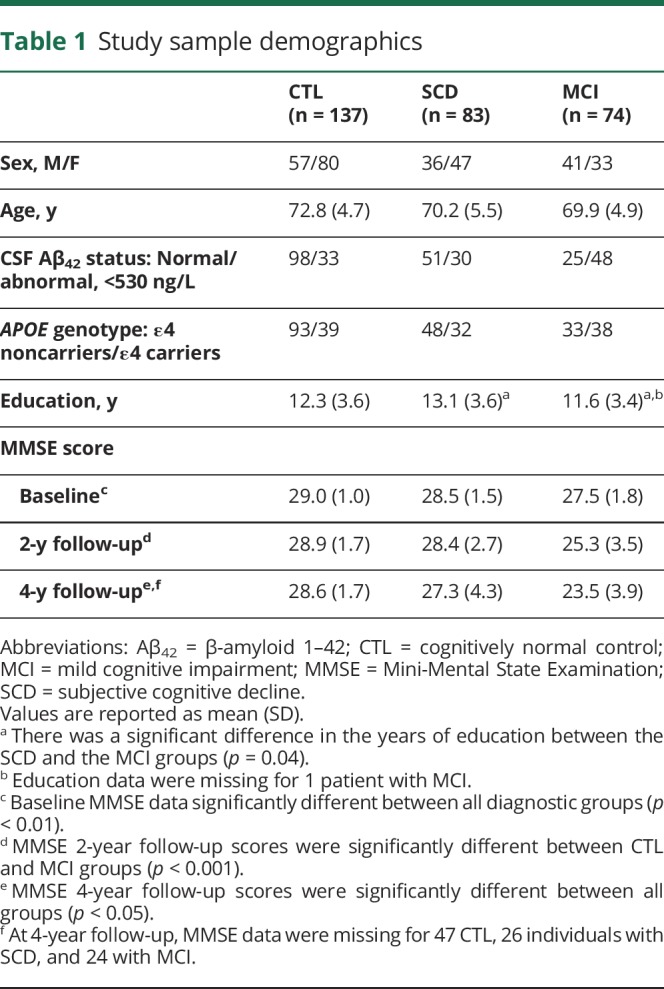
Study sample demographics

**Table 2:**
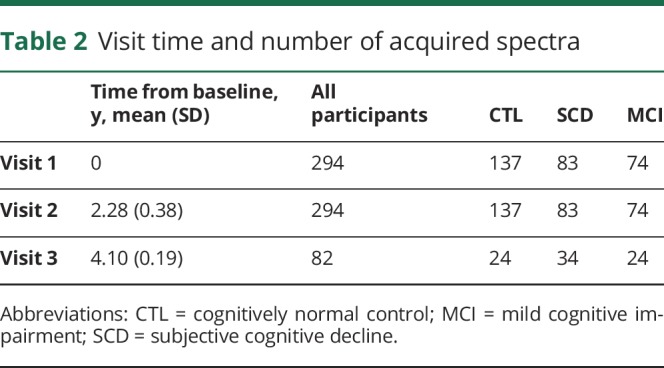
Visit time and number of acquired spectra

### Cross-sectional MRS

MRS values at baseline, visit 2, and visit 3, and cross-sectional comparisons across diagnostic groups are presented in figure 1, C and D, and data available from Dryad (Additional Results, doi.org/10.5061/dryad.rd681sp). At baseline, none of the metabolite ratios differed between diagnostic groups. On visit 2, MCI displayed higher mI/Cr and Cho/Cr values and lower NAA/mI and NAA/Cr than CTL and SCD (*p* < 0.05). On visit 3, NAA/mI remained significantly lower in MCI than CTL and SCD (*p* < 0.05), whereas mI/Cr remained higher for MCI compared to CTL (*p* < 0.05) and NAA/Cr remained lower for MCI compared to SCD (*p* < 0.05).

When we compared groups based on the individuals' Aβ status, we found no differences at baseline. At visits 2 and 3, the differences in mI/Cr, NAA/Cr, and NAA/mI were significant between Aβ-positive and -negative cases (*p* < 0.05). MRS values and cross-sectional comparisons across groups based on Aβ status are shown in data available from Dryad (Additional Results, doi.org/10.5061/dryad.rd681sp).

### Longitudinal MRS

The rate of change of MRS measures mI/Cr and NAA/mI was estimated using mixed-effect models (table 3).

**Table 3:**
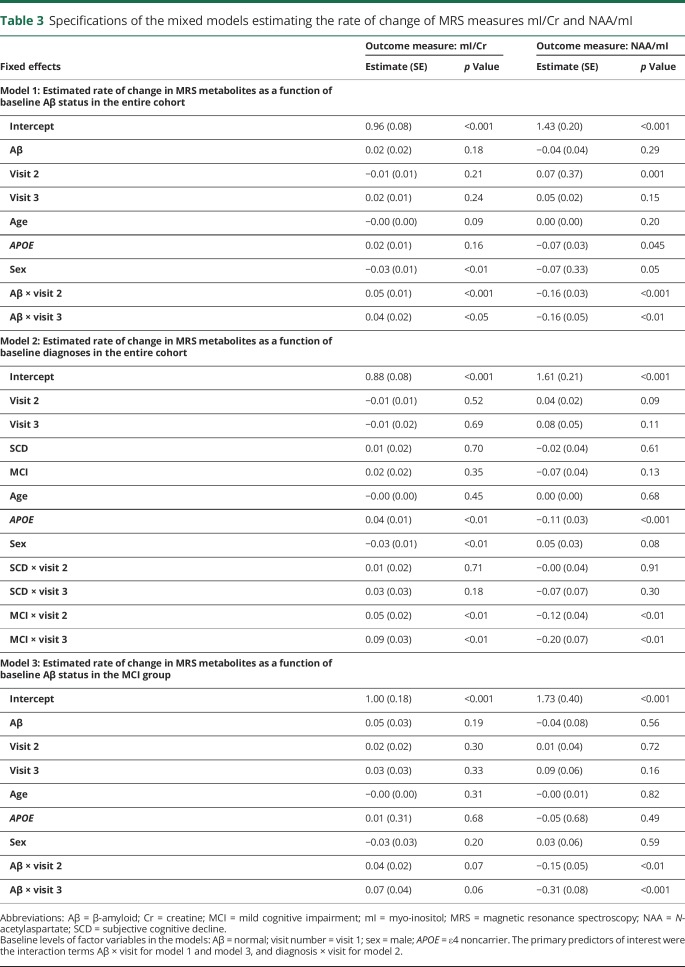
Specifications of the mixed models estimating the rate of change of MRS measures mI/Cr and NAA/mI

**Figure 2:**
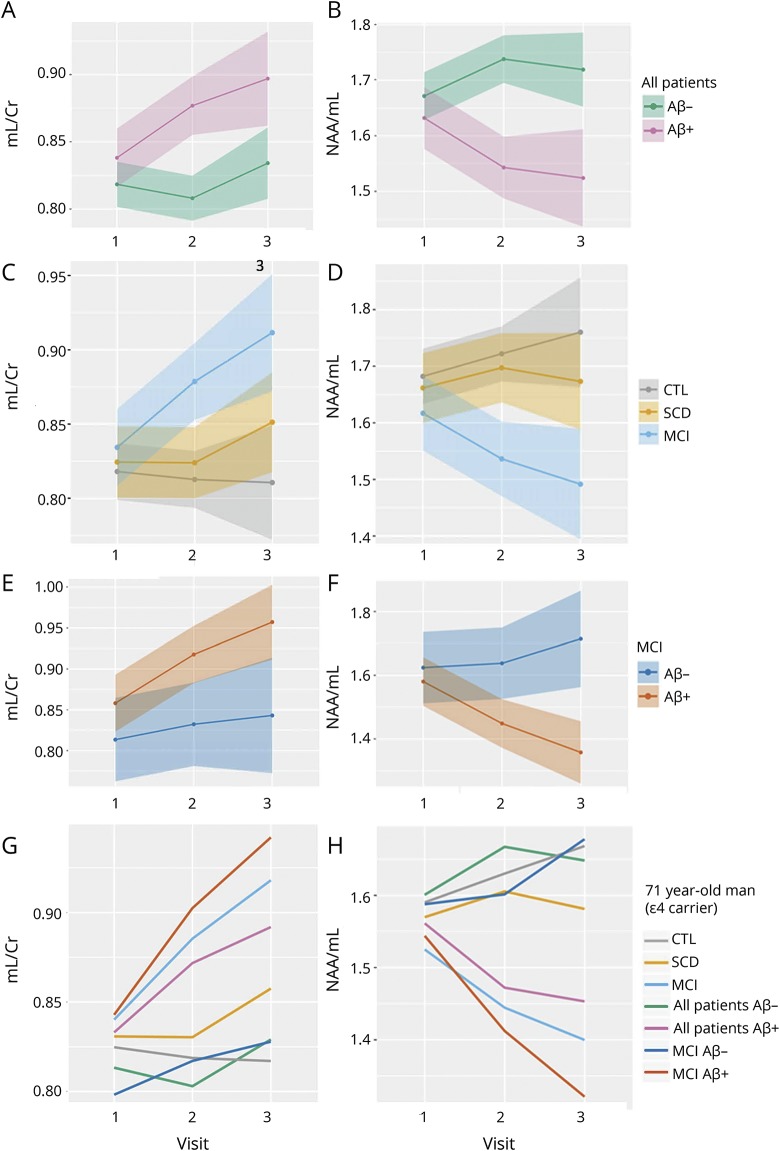
Estimated rates of change in mI measures across different biomarker and diagnostic groups Estimated rates of change in mI/Cr and NAA/mI in different biomarker and diagnostic groups. Model 1 was created using data from the entire cohort to assess whether baseline Aβ status affects the rate of change in (A) mI/Cr and (B) NAA/mI. Model 2 was built using data from the entire cohort, in order to examine whether baseline diagnosis affects the rate of change in (C) mI/Cr and (D) NAA/mI. Model 3 only used data from the MCI group as input, examining the rate of change in (E) mI/Cr and (F) NAA/mI. Based on these models, we present estimated trajectories of change over time in (G) mI/Cr and (H) NAA/mI for a 71-year-old man, who is an *APOE* ε4 carrier. Aβ = β-amyloid; Cr = creatine; CTL = cognitively normal control; MCI = mild cognitive impairment; mI = myo-inositol; NAA = *N*-acetylaspartate; SCD = subjective cognitive decline.

Model 1 was created using data from the entire cohort, in order to assess whether baseline Aβ status affects the rate of change in mI/Cr and NAA/mI, accounting for baseline age, sex, and *APOE* ε4 carriership. Thus, the primary parameter of interest for model 1 was the interaction between Aβ status and visit number. We found that although higher Aβ was not associated with higher mI/Cr levels at baseline, the estimates of longitudinal change in mI/Cr and NAA/mI were significantly different depending on the subjects' baseline Aβ status (figure 2, A and B; table 3). In the Aβ+ group, mI/Cr increased significantly with time at an estimated rate of 1.9%/y, whereas in the Aβ− group, the increase was minimal at 0.05%/y. The change in the composite ratio NAA/mI also followed significantly different trajectories over time depending on the baseline Aβ status. NAA/mI declined with the rate of 2.0%/y in the Aβ+ group. In the Aβ− group, we detected a 1.2% yearly increase in NAA/mI. However, the increase was only significant between visits 1 and 2.

Model 2 was built using data from the entire cohort, in order to examine whether baseline diagnosis affects the rate of change in mI/Cr and NAA/mI, accounting for baseline age, sex, and *APOE* ε4 carriership, irrespective of baseline Aβ status. Therefore, the primary predictor of interest for model 2 was the interaction between baseline diagnosis and visit number. The only significant change over time was found in the MCI group—here, mI/Cr increased by an average of 2.3%/y, whereas NAA/mI decreased by 2.0%/y (figure 2, C and D; table 3).

To further characterize these changes, we created model 3, using only data from the MCI group as input. As expected, the rates of change were highest in the MCI Aβ+ group at 2.93%/y (*p* = 0.07) for mI/Cr and −3.55%/y (*p* < 0.01) for NAA/mI (figure 2, E and F; table 3).

For an overview of the differences in the rates of change in mI/Cr and NAA/mI, we present the estimated MRS trajectories for a 71-year-old male *APOE* ε4 carrier for the different Aβ groups and diagnoses (figure 2, G and H).

### MRS and cognitive decline

**Figure 3:**
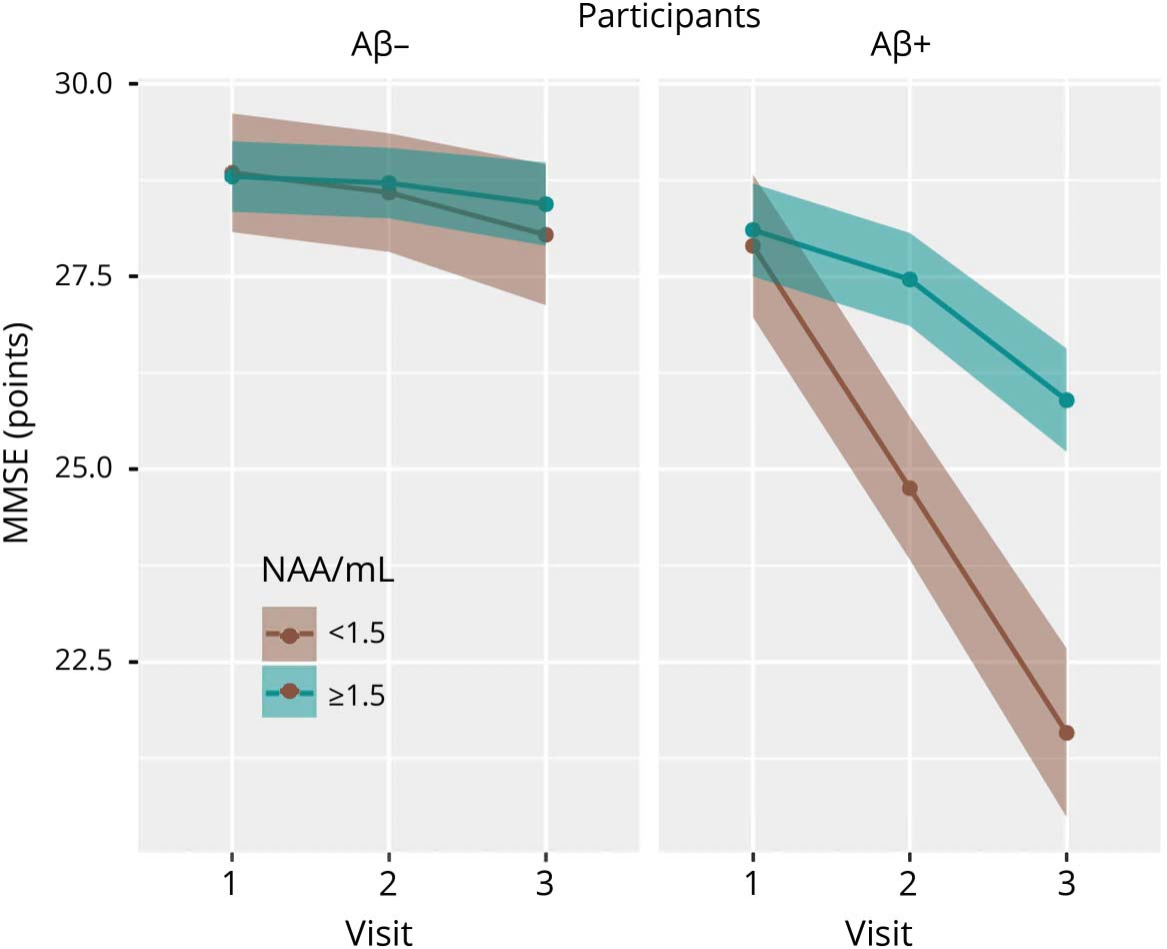
Baseline NAA/mI and cognitive decline over time Cognitive decline as a function of baseline NAA/mI. Serial MMSE data were predicted as a function of baseline NAA/mI values. We found a negligible decline in MMSE score for participants who were Aβ− at baseline. For Aβ+ participants, the trajectory of the decline in MMSE score differed significantly depending on baseline NAA/mI. Aβ+ individuals with low baseline NAA/mI (<1.5) declined by a total of 6.3 MMSE points. CSF Aβ+ individuals with high NAA/mI (≥1.5) lost a total of 2.2 MMSE points. Aβ = β-amyloid; mI = myo-inositol; MMSE = Mini-Mental State Examination; NAA = *N*-acetylaspartate.

To investigate whether baseline MRS measurements may be useful for predicting future cognitive decline, we went on to create a mixed-effects model using data from the entire cohort, where serial MMSE data were predicted as a function of baseline NAA/mI. Thus, the main predictor of interest was the interaction term between NAA/mI and visit number. We found that in participants who were Aβ− at baseline, the decline in MMSE over 4 years was negligible and effectively independent of the baseline NAA/mI. However, for Aβ+ participants, the trajectory of cognitive decline differed significantly depending on baseline NAA/mI (*p* < 0.01) (figure 3).

Individuals who were Aβ+ and had low NAA/mI at baseline (<1.5) declined by a total of 6.3 MMSE points (3.1 points between visits 1 and 2, and 3.2 points between visits 2 and 3). Individuals, who were Aβ+ at baseline, yet had higher baseline NAA/mI (≥1.5), lost a total of 2.2 MMSE points (0.6 between visits 1 and 2, and 1.6 between visits 2 and 3).

The low-NAA/mI group comprised 25 healthy controls, 22 individuals with SCD, and 22 individuals with MCI. The high-NAA/mI group comprised 6 controls, 6 SCD, and 19 MCI. The relationship between baseline MRS and longitudinal cognition was not significant when the diagnostic groups were analyzed separately.

**Figure 4:**
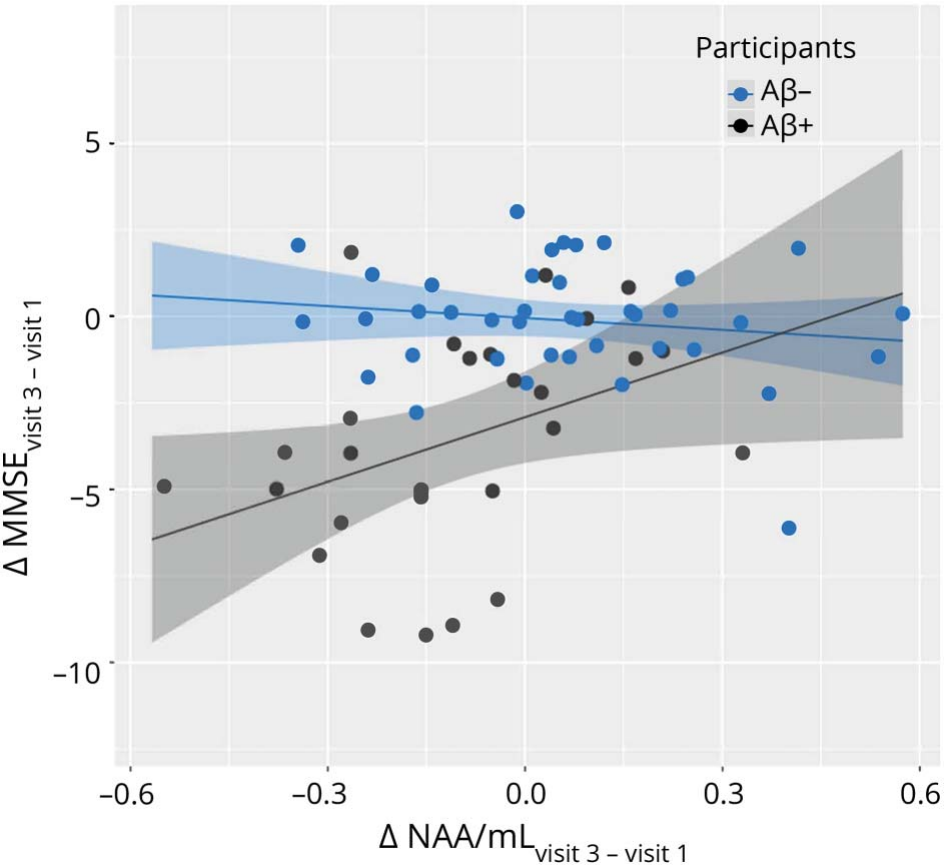
Absolute change in NAA/mI and MMSE over 4 years A larger decline in MMSE score is associated with a greater negative change (decline) in NAA/mI in Aβ+ participants but not in Aβ− participants. Aβ = β-amyloid; mI = myo-inositol; MMSE = Mini-Mental State Examination; NAA = *N*-acetylaspartate.

Furthermore, we investigated whether the absolute change in NAA/mI during the time between baseline and the last visit was associated with the absolute change in MMSE during this time. We found a significant positive association between ΔNAA/mI and ΔMMSE in the Aβ+ group but not in the Aβ− group. In a linear regression model (built using data from the Aβ+ group), where ΔNAA/mI was used to estimate the outcome ΔMMSE, accounting for sex, age, *APOE*, and education level, the variable ΔNAA/mI was found to be the only significant predictor of the ΔMMSE (*p* < 0.05). The association between ΔNAA/mI and ΔMMSE is presented in figure 4: in the Aβ+ group, a larger decline in MMSE is associated with a greater negative change (i.e., greater decline) in NAA/mI.

## Discussion

The current study highlights the link between longitudinal changes in MRS metabolites and the main pathologic hallmark of AD—amyloid accumulation. A total of 670 individual high-quality spectra were analyzed for this study—a number that far surpasses previous 3-tesla MRS study sample sizes. In general, few studies have assessed serial MRS measurements in the context of AD,^3,15–17^ of which only 2 included patients with MCI^16,17^ and none have reported information regarding amyloid pathology. Based on longitudinal MRS data from a diagnostically well-characterized cohort of 294 participants including controls and individuals with SCD and MCI, our study provides evidence of mI/Cr and NAA/mI being potentially useful markers of Aβ-related pathology. Longitudinal spectroscopic changes were evaluated in the PCC/precuneus. This region is highly involved throughout the progression of AD pathology^18–20^ and has recently been demonstrated as the earliest site for amyloid accumulation.^21^

The association between increased amyloid deposition and elevated levels of mI has previously been described cross-sectionally in vivo,^4,8^ in human tissue,^22^ and murine models.^23^ A recent study also demonstrated that higher baseline levels of mI are associated with an increased rate of Aβ accumulation over time in cognitively healthy individuals.^5^ In the present longitudinal MRS study, we expand on these findings by demonstrating that abnormal Aβ levels at baseline are predictive of elevated mI/Cr and NAA/mI concentrations over time, and examine this relationship in different clinical diagnoses. One previous multiple time-point MRS study reports that only the change in the combined ratio NAA/mI was significantly different between controls and patients with AD. The authors reported a 3.7% yearly decline of NAA/mI in patients with AD and effectively no change in controls.^24^ Although our study did not include patients with a baseline AD diagnosis, we found a similar value for the annualized change in NAA/mI in the Aβ+ MCI group (3.6% yearly decline), which is coherent with this group being most similar to the AD patient group. A 2 time-point study that included patients with MCI (but did not report NAA/mI values) found a 2.6% annual mI/Cr increase for patients with MCI subsequently converting to AD and 0% for MCI with stable cognitive function. We found a similar rate of change in the MCI group (2.3%/y). In the Aβ+ MCI group, the composite ratio NAA/mI yielded a yearly decline of 3.55%, which is comparable to the reported rates of hippocampal atrophy in MCI.^25^ Therefore, the ratio NAA/mI may be useful for monitoring disease progression, also because working with this composite marker eliminates the need for internal referencing.

The signal provided by MRS cannot explicitly determine the type of cell from which the detected resonance originates. However, since NAA is known to be synthesized exclusively in the neuronal mitochondria, there is some consensus with regard to NAA being a suitable marker of neuronal integrity and mitochondrial function.^26,27^ There is also evidence of NAA recovery as cerebral energy balance is restored in traumatic brain injury.^28^ This sensitivity to transient neuronal dysfunction probably limits the usefulness of NAA alone or NAA/Cr as a biomarker in AD. The distribution of mI among different cell types in the brain is less well understood. Generally, glial cells seem to contain higher concentrations of mI,^29,30^ but some neuronal cell lines have also been shown to contain high mI levels.^31^ Although brain mI levels are elevated in a number of conditions associated with gliosis, mI can also increase in absence of glial activation.^32^ One of the most important findings of the current study is the substantial difference observed in the rate of mI increase between the Aβ− and Aβ+ groups. Several mechanisms (independent or interlinked) may be responsible for this phenomenon. A known physiologic role of mI is that of an organic osmolyte^33^; intracellular mI concentrations increase as a response to extracellular hypertonicity^34^ and decrease in hypotonic conditions.^35^ Thus, it is possible that the longitudinal accumulation of mI in the Aβ+ detects osmoregulation—an effort to resist homeostatic aberrations associated with pathology. A recent study demonstrated that mI accumulation in response to hypertonic stress is able to disturb electrical properties of excitable cells,^36^ suggesting that increase in mI observed in our study may be linked to synaptic dysfunction independently of Aβ. However, there is convincing evidence of a relationship between Aβ plaque pathology and mI. Brain mI is elevated at predementia stages of Down syndrome^37^ and familial AD,^38^ and antemortem mI levels are associated with postmortem cored and diffuse amyloid plaques.^23^ Furthermore, removal of Aβ plaques attenuates mI levels in APP-PS1 mice.^39^ A recent retrospective, longitudinal MRS study found that individuals who had progressed to AD at 7-year follow-up already had higher mI/Cr levels at baseline than those who had remained stable.^40^ It is therefore probable that the increase in mI/Cr in the Aβ+ group observed in this study is indicative of the ongoing accumulation of cortical Aβ burden within this group. A serial amyloid PET study performed in conjunction with serial MRS would confirm this hypothesis.

It has been shown that individuals with high baseline load of Aβ are at risk of a steeper cognitive decline.^41^ Also, there is recent evidence suggesting that MRS measurements have the potential to identify individuals at risk of a more rapidly progressing AD pathology.^5^ This evidence of an interplay between MRS concentrations, amyloid accumulation, and cognition led us to explore the potential of baseline mI/NAA to predict the trajectory of future cognitive decline. Our results demonstrate that in the entire sample, Aβ+ individuals with low baseline NAA/mI display a steeper cognitive decline trajectory, losing on average 4 more MMSE points in total than those with higher baseline NAA/mI levels. Of interest, this apparent ability of MRS metabolites to predict the extent of future decline was only evident in the subgroup of individuals who were Aβ+ at baseline. We demonstrate that additional differentiation of Aβ+ individuals based on an MRS threshold may reveal subgroups with markedly different rates of cognitive decline over time. The composite ratio NAA/mI has previously been demonstrated to be a good predictor of an AD diagnosis.^16,40^ Our finding expands on this knowledge by demonstrating that NAA/mI may be useful for predicting the extent of cognitive changes (one aspect of disease severity) at an earlier disease stage. The composite ratio of NAA and mI in AD is emerging as the most useful MRS marker in AD since this measure may incorporate 2 of the main disease hallmarks—amyloid pathology and neurodegeneration.

Although our findings support the assumption of the existence of an AD-specific MRS metabolic signature, it is important to recognize that this study does not aim to evaluate the usefulness of MRS as a marker of Aβ positivity, nor does it assess the ability of MRS alone to predict cognitive decline in the general population. Rather, we highlight the value in combining CSF and MRS data and demonstrate that MRS may provide new information regarding the rate of future cognitive decline once amyloid positivity has been established. The nature of our study population is such that the existence of comorbidities cannot be ruled out. It is therefore possible that some of the alterations in the MRS profile may be due to other causes. Although abnormal CSF amyloid measures provide a good indication of ongoing AD pathology, in an ideal setting, information about the timing and the number of individuals who convert to AD should also be used. Furthermore, the lack of normative spectroscopic data, or a clear understanding of the biological processes causing MRS alterations, corroborate the need for CSF measures in order to interpret any MRS changes.

The design of our study was such that a cutoff value has been used to stratify participants into Aβ− and Aβ+ based on baseline CSF Aβ_42_ levels. Furthermore, one of our results suggests that there may exist some cutoff value for NAA/mI that is relevant for predicting a worsening of cognitive symptoms. There are certain disadvantages to working with dichotomized biomarkers, such as the possible masking of subthreshold effects and the assessment of individuals close to the cut point. Nevertheless, applying a normal/abnormal biomarker classification is an approach that, apart from being practical, is essential for determining eligibility for clinical trials.^42^ However, we recognize that a putative cutoff value for NAA/mI, which may have relevance for determining an individual's cognitive decline profile, must be scrutinized by future studies and validated in even larger longitudinal cohorts.

The inclusion criteria of the BioFINDER cohort and the final constellation of our study sample may limit the generalizability of the results of this study. The potential sources of selection bias in the recruitment of the participants to the BioFINDER may stem from the relatively high cognitive scores at entry and the requirement to undergo a lumbar puncture and an MRI. However, although study participants might be healthier than the general population, it is unclear in which direction this would affect the associations found in this study.

In conclusion, our findings demonstrate that the longitudinal changes in mI/Cr and NAA/mI are largely governed by the presence of underlying amyloid pathology, warranting their potential usefulness as noninvasive dynamic disease biomarkers during the predementia stages of AD. Today, amyloid and tau deposition can be imaged using PET, allowing us to assess molecular pathology in vivo. However, the need remains for a more widely available cost-effective technique that can be used for screening dementia and monitoring disease progression and treatment effects in a clinical setting. Large-scale multimodal studies that include MRS will help further locate spectroscopic changes in the continuum of AD pathophysiologic processes.

## Glossary

Aβ: β-amyloid
AD: Alzheimer disease
BioFINDER: Biomarkers for Identifying Neurodegenerative Disorders Early and Reliably
Cho: choline
Cr: creatine
CTL: cognitively normal control
MCI: mild cognitive impairment
mI: myo-inositol
MMSE: Mini-Mental State Examination
MRS: magnetic resonance spectroscopy
NAA: *N*-acetylaspartate
PCC: posterior cingulate cortex
SCD: subjective cognitive decline
